# Brain network functional connectivity changes in patients with anterior knee pain: a resting-state fMRI exploratory study

**DOI:** 10.1186/s41747-023-00378-1

**Published:** 2023-10-09

**Authors:** Vicente Sanchis-Alfonso, María Beser-Robles, Amadeo Ten-Esteve, Cristina Ramírez-Fuentes, Ángel Alberich-Bayarri, Raúl Espert, Luis García-Larrea, Luis Martí-Bonmatí

**Affiliations:** 1 Department of Orthopedic Surgery, Hospital Arnau de Vilanova, Valencia, Spain; 2 Biomedical Imaging Research Group (GIBI230), Hospital Universitario Y Politécnico E Instituto de Investigación Sanitaria La Fe, Valencia, Spain; 3 Department of Technologies for Health and Well-Being, Polytechnic University of Valencia, Valencia, Spain; 4 Radiology Department, Hospital Universitario Y Politécnico La Fe, Valencia, Spain; 5 Quantitative Imaging Biomarkers in Medicine, QUIBIM SL, Valencia, Spain; 6 Departamento de Psicobiología, Unidad de Neuropsicología, Hospital Clinic Universitari, Universidad de Valencia, Valencia, Spain; 7 Center for Neuroscience Research of Lyon (CRNL), NeuroPain Team, U 1028, INSERM, Lyon-1 University, Bron, France

**Keywords:** Chronic pain, Brain, Catastrophization, Knee joint, Magnetic resonance imaging

## Abstract

**Background:**

This study investigates the functional brain connectivity in patients with anterior knee pain (AKP). While biomechanical models are frequently employed to investigate AKP, it is important to recognize that pain can manifest even in the absence of structural abnormalities. Leveraging the capabilities of functional magnetic resonance imaging (fMRI), this research aims to investigate the brain mechanisms present in AKP patients.

**Methods:**

Forty-five female subjects (24 AKP patients, 21 controls) underwent resting-state fMRI and T1-weighted structural MRI. Functional brain connectivity patterns were analyzed, focusing on pain network areas, and the influence of catastrophizing thoughts was evaluated.

**Results:**

Comparing patients and controls, several findings emerged. First, patients with AKP exhibited increased correlation between the right supplementary motor area and cerebellum I, as well as decreased correlation between the right insula and the left rostral prefrontal cortex and superior frontal gyrus. Second, in AKP patients with catastrophizing thoughts, there was increased correlation of the left lateral parietal cortex with two regions of the right cerebellum (II and VII) and the right pallidum, and decreased correlation between the left medial frontal gyrus and the right thalamus. Furthermore, the correlation between these regions showed promising results for discriminating AKP patients from controls, achieving a cross-validation accuracy of 80.5%.

**Conclusions:**

Resting-state fMRI revealed correlation differences in AKP patients compared to controls and based on catastrophizing thoughts levels. These findings shed light on neural correlates of chronic pain in AKP, suggesting that functional brain connectivity alterations may be linked to pain experience in this population.

**Relevance statement:**

Etiopathogenesis of pain in anterior knee pain patients might not be limited to the knee, but also to underlying alterations in the central nervous system: cortical changes might lead a perpetuation of pain.

**Key points:**

• Anterior knee pain patients exhibit distinct functional brain connectivity compared to controls, and among catastrophizing subgroups.

• Resting-state fMRI reveals potential for discriminating anterior knee pain patients with 80.5% accuracy.

• Functional brain connectivity differences improve understanding of pain pathogenesis and objective anterior knee pain identification.

**Graphical Abstract:**

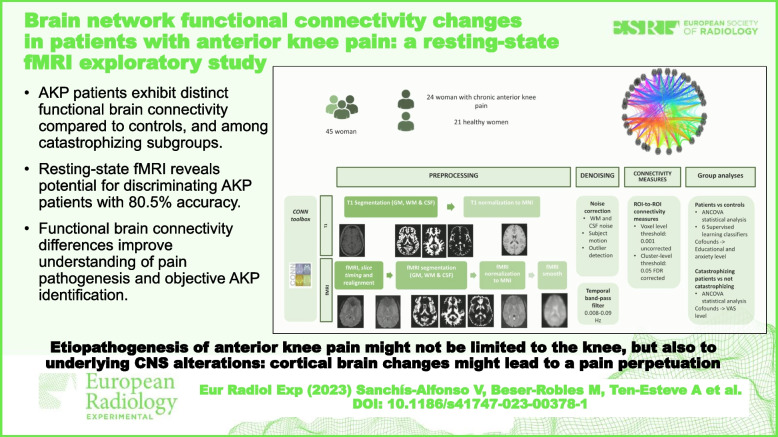

## Background

Anterior knee pain (AKP), defined as “pain around or behind the patella, which is aggravated by at least one activity that loads the patellofemoral joint during weight-bearing on a flexed knee (*e.g.*, squatting, stair ambulation, jogging/running, hopping/jumping)” [1], is the most frequent reason why active young females consult with a knee orthopedic surgeon [2]. A peculiar characteristic of this condition is the large variability among patients regarding the magnitude of pain and pain experience [2]. Moreover, there is a great variability in the response to treatment [2].

In spite of its high incidence and abundance of clinical research, its pathogenesis is not clearly understood [3, 4]. It is widely accepted that structural anomalies cannot fully account for pain and disability in these patients as there is a poor correlation between structural alterations of the patellofemoral joint with pain and disability [5, 6]. Pain etiology in AKP patients is multifactorial, with many contributors, so structural changes or any other factor alone are unlikely to explain pain. Signs of central sensitization have been shown in individuals with AKP, indicating altered pain modulation [7]. Moreover, growing evidence suggests that psychological features play a role in persistent musculoskeletal pain. Factors such as anxiety, depression, catastrophizing thoughts and kinesiophobia have been associated with AKP and disability [8–10]. Among all the psychological factors, the most relevant one from a clinical standpoint is catastrophizing [8].

Functional magnetic resonance imaging (fMRI) techniques have recently provided insights into the mechanisms underlying the development of chronic pain, and AKP is a paradigm of chronic pain, identifying pain centers that work as networks. This is done by studying the structural and functional connectivity (FC) of brain networks, measuring neural activity through blood oxygenation level-dependent (BOLD) activity. “Pain neuromatrix” has been defined as a group of brain regions that are activated together in a coordinated manner by painful stimuli, being considered the basis of the multidimensional experience of pain [11, 12]. In the study of this neuromatrix, Kucyi et al. [11] highlighted the importance of thalamus, somatosensory cortex, insular cortex, prefrontal cortex, anterior cingulate cortex, lentiform nucleus, cerebellum, amygdala, and nucleus accumbens. Kucyi et al. also described the main brain networks related to the chronicity of pain such as the salience network (SN), the default mode network (DMN) and the sensorimotor network (SMN). DMN seems to play a key role in the synchronization of all brain regions [13]. Other functional networks, such as limbic, attentional, central executive network (CEN), and frontoparietal network, are also active during the processing of pain stimuli [14, 15].

The primary purpose of this study was to define FC changes in AKP patients compared with matched controls and between AKP patients with catastrophizing thoughts and AKP patients without catastrophizing thoughts. The objective of this study is to investigate the impact of chronic AKP on brain dynamics at rest. We hypothesized that chronic AKP may be associated with fMRI brain changes in interconnected networks, which may modulate the variable impairments accompanying this condition, and even explain the different responses to treatment. The second objective of this study was to evaluate the accuracy of fMRI to assess AKP.

## Methods

### Participants and clinical assessments

A total of 45 subjects were recruited and evaluated, 24 women with chronic AKP of ≥ 6 months duration, ranging in age from 18 to 44 years (27.9 ± 9.0 years, mean ± standard deviation), and 21 healthy women without knee pain, ranging in age from 20 to 43 years (28.7 ± 7.5 years). Patients and controls were age-matched. All patients are females because AKP is a pathological entity typical of women. In addition, the fact that they are all females avoids bias in our results. In this way, Silberstein and Camfield [16] observed that the functional brain networks involved in vigilance tasks are different in males and females. The study was approved by the hospital's Institutional Review Board (CEIm:3/2018) and was conducted in accordance with the Declaration of Helsinki. All subjects gave written consent for this study. Inclusion criteria for the AKP group included patients with self-reported pain lasting at least 6 months. Exclusion criteria for the control group included acute or chronic pain within the last 6 months and history of psychological or psychiatric disorders.

Demographic variables recorded were age, dominant hand, schooling, and levels of depression and anxiety. All patients completed a booklet with self-report questionnaires on pain intensity, anxiety, depression, kinesiophobia, and catastrophizing. Clinical pain intensity was obtained by asking subjects to rate their pain on a visual analog scale (VAS) [17]. Anxiety and depression were evaluated using the Hospital Anxiety and Depression Subscale [18], with a value greater than 11 was considered as anxious or depressive. Pain-related fear associated with avoidance of movement and physical activity was measured using the Tampa Scale for Kinesiophobia [19], and catastrophizing was measured using the Pain Catastrophizing Scale [20].

### Resting-state fMRI data acquisition and analysis

MRI data were acquired on a 3-T scanner (Achieva TX, Philips Healthcare Best, The Netherlands) using an 8-channel head coil with parallel acquisition technology (SENSE). All participants were instructed at the beginning of the acquisitions to avoid movements, keep their eyes closed, stay awake and think of a blue sky. The acquisition protocol consisted of a high spatial resolution T1-weighted three-dimensional gradient-echo sequence with the following parameters: echo time 3,000 ms; repetition time 6,200 ms; flip angle 10^0^; voxel size 1 × 1 × 1 mm^3^; and duration of 6 min. The resting-state fMRI T2*-weighted two-dimensional echo-planar BOLD sequence was acquired with the following main parameters: echo time 35 ms; repetition time 2,000 ms; 256 temporal dynamics (corresponds to the number of temporal instants acquired in this acquisition. Each of these dynamics represents a point in time during the imaging sequence and contains information about brain activity at that specific time); pixel size 1.8 × 1.8 mm^2^; slice thickness 5 mm; and overall duration 9 min. This sequence allows to explore networks FC by sampling the brain hemodynamic response during neuronal activation at the resting-state.

All images were preprocessed using the CONN [21] and SPM12 [22] toolboxes. Resting-state fMRI images were corrected (intra-patient registration) from slice time and patient movement, normalized to the Montreal Neurological Institute (MNI) space, registered with the structural images and smoothed. Artefact detection was used to depict intensity peaks and excessive patient movements by using ART-repair software [23] and a component-based noise correction method (CompCor [24]). For excessive patient movements, acquisitions showing a mean image shift greater than 0.9 mm or global BOLD signal changes greater than 5 standard deviations were flagged as possible outliers.

The intensity level of BOLD time-series z-score was normalized and images were spatially registered towards a standardized MNI space. Segmentation, (separation into gray matter, white matter, and cerebrospinal fluid), was applied. Then, brain parcellation was performed using the Functional Magnetic Resonance Imaging of the Brain, FMRIB, Software Library Harvard–Oxford probabilistic atlas, which provides 91 cortical and 15 subcortical regions of interest (ROIs) plus 26 cerebellar regions defined by the Automated Anatomical Labelling, AAL, atlas. Six commonly characterized networks were obtained by seed-areas on known networks: SN, DMN, Dorsal Attention Network (DAN), SMN, Visual Network (VN), and Cerebellar Network (CN).

The DMN comprises the medial prefrontal cortex, parietal lobe, and posterior cingulate cortex, and is mainly activated during introspection. On the other hand, the SMN is composed of the basal ganglia, thalamus, posterior insula, and somatosensory cortex, and is essential for body sensation awareness and the generation of appropriate motor responses. Additionally, the SN is formed by the anterior cingulate cortex, anterior insula, rostral prefrontal cortex, and the supramarginal gyrus. This network is responsible for processing sensory stimuli and generating affective responses. The VN is constituted by the cuneal cortex, intracalcarine cortex, occipital fusiform cortex, and lingual gyrus. The DAN is formed by the frontal eye field and the intraparietal sulcus, which are crucial for attentional processes. Finally, the anterior and posterior cerebellar areas form the CN. Each of these networks plays a distinct and vital role in the brain's functioning and interactions, contributing to various cognitive functions and perceptual processes.

On the other hand, the CEN is a network that encompasses some of the areas described in these networks, such as the dorsolateral prefrontal cortex, ventrolateral prefrontal cortex, anterior cingulate cortex, anterior insula, motor supplement area, and posterior parietal cortex. This network is a fundamental part of the system of brain networks involved in cognitive processing and control in the human brain.

Before image processing, main confounder effects (24 parameters for head movement obtained from the ART-repair program and intensity effects that do not correspond to the gray matter) were included in a linear regression model, with a 0.008–0.09 Hz bandpass filtering to obtain BOLD time-series signal free of unwanted effects.

The ROI-to-ROI analysis, atlas-based, was performed representing the level of partial correlation between all the pairs of brain ROIs. Using a general linear model to estimate the strength of connectivity between brain areas, using a partial correlation analysis between the averaged BOLD signal from each pair of brain ROIs. Conducting two different group analyses: AKP patients *versus* controls, and catastrophizing patients *versus* patients without catastrophizing. Effect sizes represent Pearson correlation coefficients (*r*) with a Fisher's *z*-transformation, which are represented as ß coefficients.

In the statistical analysis, significant clusters were determined by two thresholds, one at connection level and one at cluster level. The significance level was defined by a connection-level threshold of *p* < 0.001 uncorrected to control for cluster spread, and a cluster-level threshold of *p* < 0.05 corrected for false discovery rate (FDR), for multiple comparisons across the whole brain [25]. The connection-level threshold was used to identify individual connections that exhibited significant effects within the clusters identified during the cluster-level analysis. This analytical approach allowed us to explore specific connections within brain networks that contribute to the observed differences between patients with anterior knee pain and healthy controls.

The first analysis was a between-group comparison to discover significant unbiased resting-state fMRI differences between patients and controls, by means of an ANCOVA statistical analysis adding as covariates the variables that show significant differences between patients and controls, with the aim of making sure that these differences are due to FC and not to these. The second analysis compared the FC of patients with catastrophizing *versus* those without, following the same statistical model as in the previous analysis.

Finally, we explored the potential of a multivariate classification using the value of the correlation between the regions we found significant in the ANCOVA analysis to differentiate AKP patients from controls. These correlations were extracted from the ROI.mat matrix resulting from the CONN software. For this purpose, 6 supervised learning classifiers were used.

The supervised classification approach allowed us to utilize the correlation between brain regions, to predict the group of each patient. We employed a set of well-established supervised learning classifiers to perform the classification task. The chosen classifiers were logistic regression, k-nearest neighbors, decision tree, linear discriminant analysis, support vector classification, and Gaussian naive Bayes. These classifiers were implemented using the Scikit-learn library, a widely-used machine learning library in Python [26].

To assess the performance of the classifiers, we employed the k-fold cross-validation method. We divided the sample into ten subsets and conducted k iterations, with each iteration using one subset for testing and the remaining subsets for training. This approach allowed us to robustly evaluate the classifiers' performance across different training and testing sets. We evaluated the models based on two common metrics: accuracy and the area under curve at receiver operating characteristics analysis (ROC-AUC) Accuracy measures the proportion of correctly classified samples, providing an overall performance measure. The ROC-AUC represents the classifier's ability to discriminate between the AKP and control groups, with a higher ROC-AUC indicating a better discriminatory power.

**Fig. 1:**
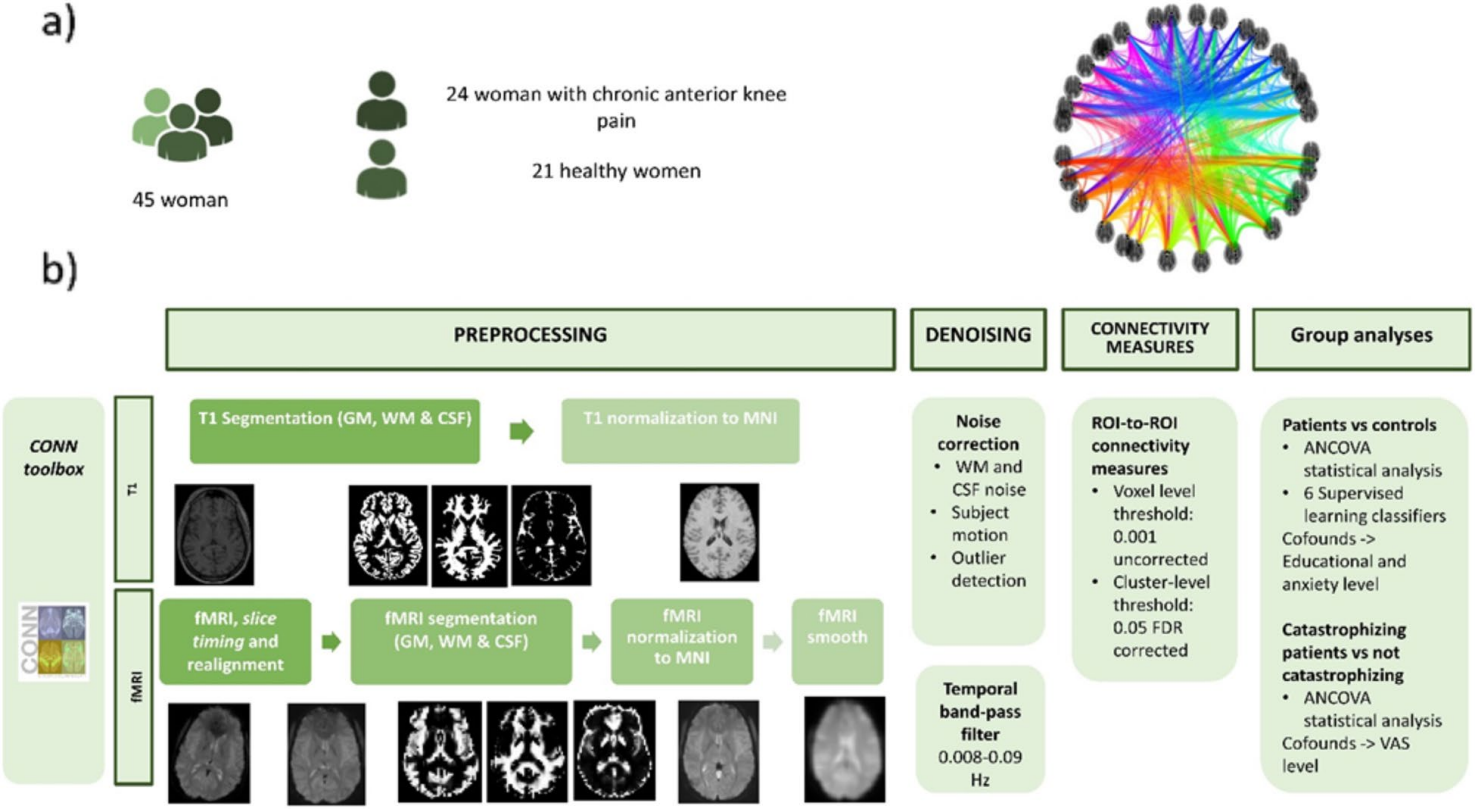
Scheme of methodology followed in this study. **a** shows the separation of the sample between patients and controls while **b** shows the flow followed to perform the analysis, including preprocessing, denoising, connectivity analysis and cluster analysis. *GM* Gray matter, *WM* White matter; *CSF* Cerebrospinal fluid, *MNI* Montreal Neurological Institute atlas, *fMRI* Functional magnetic resonance imaging, *ANCOVA* Covariance analysis, *VAS* Visual analogue scale

The methodology followed in this study, including the CONN steps of preprocessing, denoising and connectivity measures implementation of supervised classification models and the evaluation using k-fold cross-validation and accuracy/AUC metrics, is illustrated in Fig. 1.

## Results

The demographic variables collected show significant differences between patients and controls in the level of schooling and level of anxiety (Table 1). As for the variables related to pain, we see that the mean VAS in patients is 6.85 ± 1.70 and a large proportion of patients show results compatible with kinesiophobia (75%) and catastrophizing thoughts (58%). According to the level of catastrophizing we only found significant differences in the variable VAS.

**Table 1:** Demographic variables recorded

Variable	Category	AKP patients	Controls	*χ*^2^	*p* value	Catastrophizing patients	Non-catastrophizing patients	*χ*^2^	*p* value
Age (years)	−	27.9 ± 8.9	28.7 ± 7.5	−	0.700	28.2 ± 8.1	27.4 ± 10.5	−	0.800
Dominant hand	Left-handed	15%	9.5%	0.3	0.600	9.1%	22.2%	0.7	0.400
	Right-handed	85%	90.5%			90.9%	77.8%		
Education	Secondary studies	70.8%	100%	7.3	0.007	72.7%	88.9%	0.8	0.400
	Primary studies	29.2%	0%			27.3%	11.1%		
Anxiety	Non-anxious	70%	100%	5.2	0.023	63.6%	77.8%	0.5	0.500
	Anxious	30%	0%			36.4%	22.2%		
Depression level	Non-depressive	90%	100%	2.2	0.100	81.8%	100.0%	1.8	0.200
	Depressive	10%	0%			18.2%	0.0%		
VAS level	−	6.85 ± 1.70	−	−	−	7.6 ± 1.2	5.8 ± 1.7		0.005
Kinesiophobia	Non-kinesiophobic	0% 75%	−	−	−	9.1%	44.4%	3.3	0.100
						90.9	55.6%		
	Kinesiophobic								

Continuous variables are expressed as mean and standard deviation, categorical variables as percentages. A two-sample Student *t*-test and a *χ*^2^ test were applied for testing differences between patients and controls and between catastrophizing and non-catastrophizing patients, taking a significance level *p* value < 0.05. *AKP* Anterior knee pain, *VAS* Visual analogue scale

**Fig. 2:**
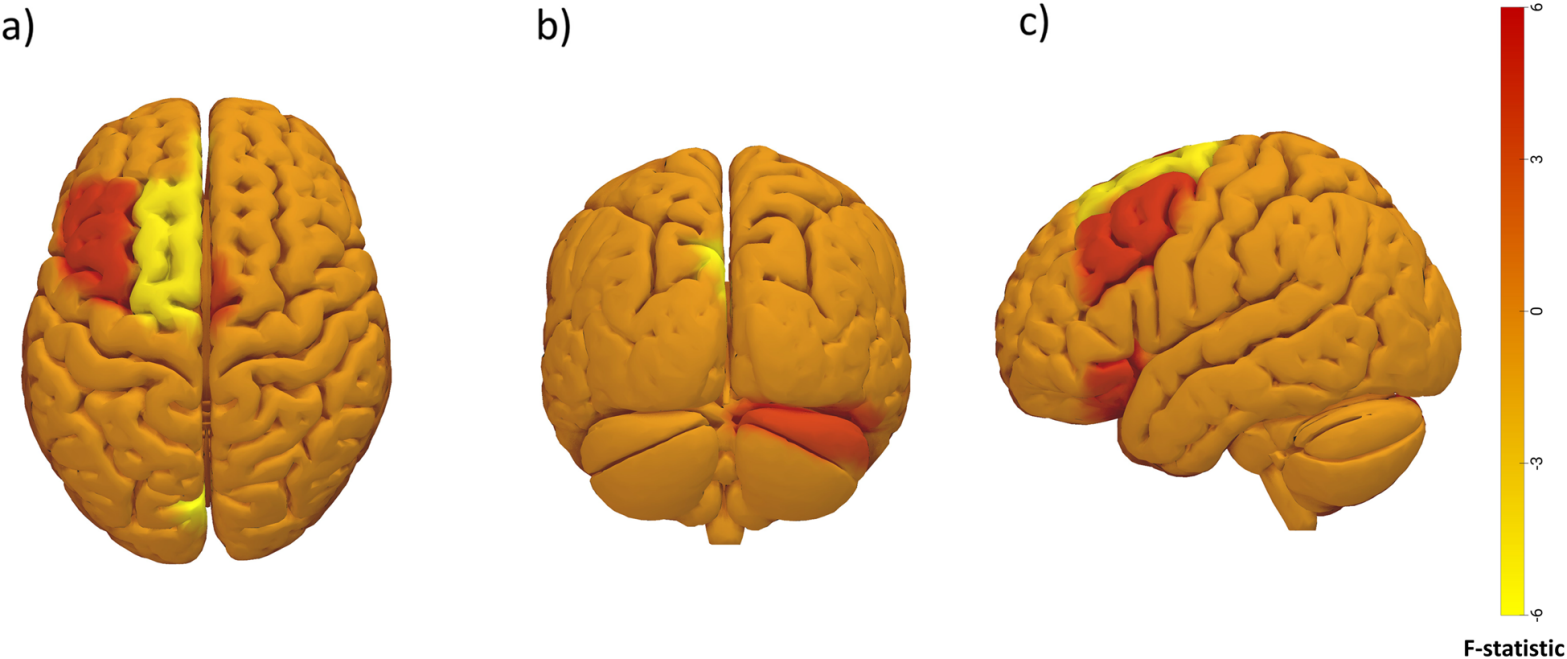
CONN output figure highlighting the clusters showing group differences between patients and controls, mentioned in the Table 2. Specifically, connections with higher positive *F* statistic values are depicted in red, while connections with higher negative *F* statistic values are represented in yellow colors. The color bar represents the *F* distribution value

Significant correlation differences between regions (*p*-FDR < 0.05) were found between AKP patients and matched healthy controls (Table 2 and Fig. 2). For most significant results, a lower correlation was observed between patients’ ROIs compared to controls, except for superior frontal gyrus area with insula, where a higher correlation was observed in AKP patients. Of all the results, we highlight the correlation changes produced in areas of the SN within it and with CEN areas. This network has a very important role in pain stimuli processing. On the other hand, we highlight the role of the cerebellum, showing an increase in correlation between its anterior part and areas of the SMN and a decrease between its posterior part and visual areas. To characterize the nature of relative FC changes between patients and controls, mean *z*-scores were extracted from significant clusters. This revealed that patients exhibited enhanced anticorrelation (negative FC) rather than less positive FC.

**Table 2:** Functional connectivity AKP patients *versus* controls

ROI 1	MNI coordinates	ROI 2	MNI coordinates	ß patients	ß controls	F distribution	*p*-FDR *p* < 0.05	Correlation strength
Cuneal l	(-13, -76, 35)	Posterior cerebellar network	(0, -79, -32)	-0.36	0.11	-5.36	0.001	↓ in patients
Anterior Insular cortex r	(47, 14, 0)	Superior frontal gyrus l	(-15, 59, 20)	-0.70	0.35	-3.90	0.039	↓ in patients
		Rostral prefrontal cortex l	(-32, 45, 27)	-0.30	0.03	-3.81	0.039	↓ in patients
Rostral prefrontal cortex l	(-32, 45, 27)	Middle frontal gyrus l	(45, 53, -7)	0.28	-0.26	4.61	0.007	↑ in patients
Supplementary motor area r	(-2, -4, 58)	Cereb1 r	(14, -58, -24)	0.13	-0.25	4.01	0.043	↑ in patients

ROI1 and ROI2 constitute the pair of evaluated regions on which the resting-state fMRI functional connectivity has shown significant differences between patients and controls. ß Patients represents the patient’s correlation between ROI1 and ROI2, while ß controls is the control correlation. Columns 2 and 4 indicate the spatial location of the regions in the Montreal Neurologic Institute (MNI) coordinates. The correlation strength represents the behavior of the correlation between the two ROIs, whether it increases or decreases in the case of patients. *F* distribution is the value that represents the ratio of variances of the patient and control groups. *FDR* False discovery rate, *fMRI* Functional magnetic resonance imaging, *ROI* Region of interest

**Fig. 3:**
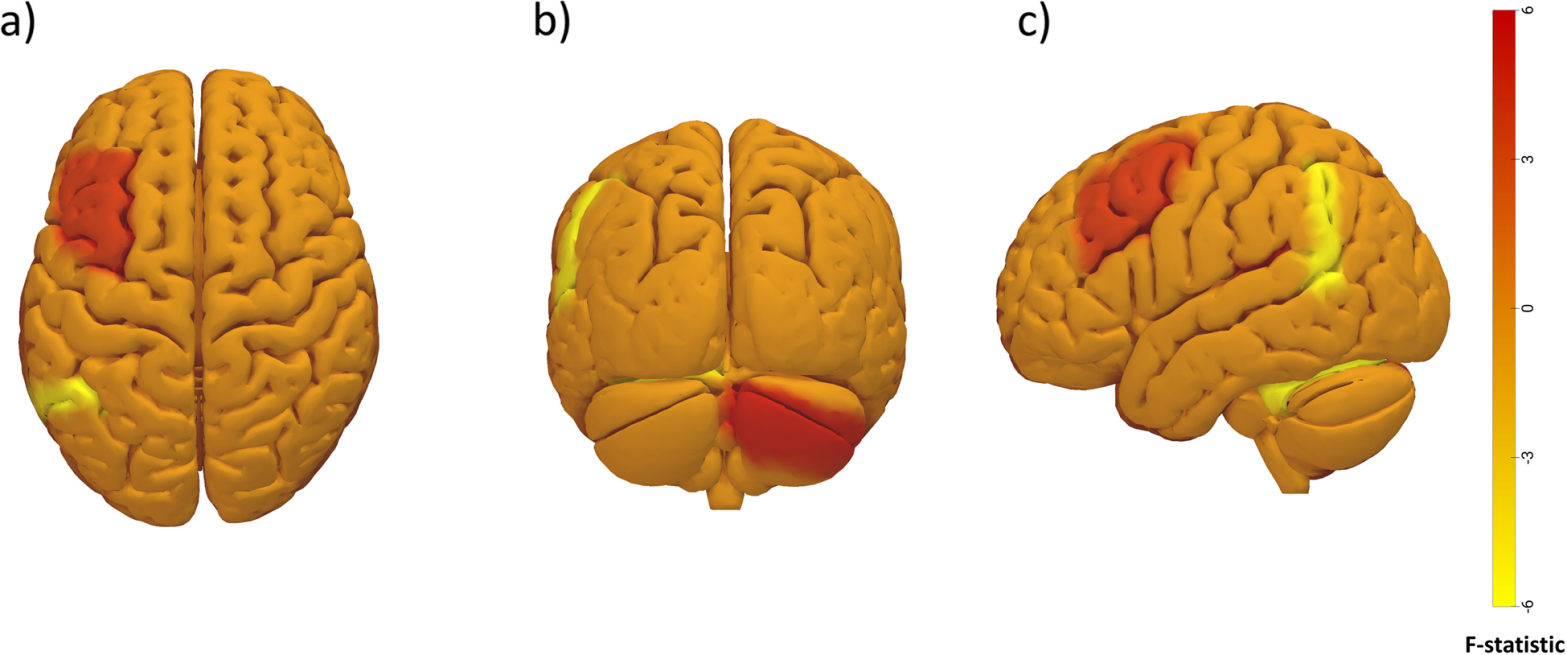
CONN output figure highlighting the clusters showing group differences between catastrophizing and non-catastrophizing patients, mentioned in Table 3. Specifically, connections with higher positive *F* statistic values are depicted in red, while connections with higher negative *F* statistic values are represented in yellow colors. The color bar represents the *F* distribution value

Subsequent analysis of all subjects to assess the effect of catastrophizing ideas also showed statistically significant differences (*p*-FDR < 0.05) in FC (Table 3 and Fig. 3). In this analysis for most FC changes, we observed a higher correlation in those patients showing catastrophizing thoughts in areas of the DMN with areas of the cerebellum and limbic areas. Alterations were observed in areas of the CEN within it and with areas of the SN. In this analysis, we again tested the nature of the changes in FC and found that decreases in correlation corresponded to greater anti-correlation between regions.

**Table 3:** Functional connectivity AKP Catastrophizing subjects

ROI 1	MNI coordinates	ROI 2	MNI coordinates	ß catastrophizing	ß non-catastrophizing	F distribution	*p*-FDR	Correlation strength
Lateral Parietal l	(-39, -77, 33)	Palidum r	(22, 6, -2)	0.31	-0.43	3.85	0.047	↑ with catastrophizing
		Cereb2 r	(22, -88, -38)	0.44	-0.86	5.33	0.039	↑ with catastrophizing
		Cereb7 r	(18, -40, -54)	0.19	-0.95	4.44	0.017	↑ with catastrophizing
Middle frontal gyrus l	(44, 34, 20)	Dorsolateral prefrontal cortex r	(41, 38, 30)	0.27	-0.41	4.14	0.047	↑ with catastrophizing
		Thalamus r	(15, -24, 15)	-0.31	0.25	-4.05	0.047	↓ with catastrophizing
Angular gyrus l	(48, 64, 30)	Cereb6 l	(-33, -59, -21)	-0.33	0.55	-4.61	0.022	↓ with catastrophizing

ROI1 and ROI2 constitute the pair of evaluated regions on which the resting-state fMRI functional connectivity has shown significant differences between patients and controls. ß represents the catastrophizing patient’s correlation between ROI1 and ROI2. Columns 2 and 4 indicate the spatial location of the regions in the Montreal Neurologic Institute (MNI) coordinates. The correlation strength represents the behavior of the correlation between the two ROIs, whether it increases or decreases in the case of catastrophizing patients. F distribution is the value that represents the ratio of variances of the catastrophizing and non-catastrophizing patient groups. *FDR* False discovery rate, *fMRI* Functional Magnetic resonance imaging, ROI Region of interest

The results of significant correlations between regions and networks of AKP patients were used as inputs to feed the classifiers. After testing the six supervised learning classification models, the decision tree model was chosen as having the best accuracy (0.805) and ROC-AUC (0.821) (Table 4).

**Table 4:** Accuracy and ROC-AUC classifier results for each supervised learning model

Supervised learning model tested	Accuracy (%)	ROC-AUC
Logistic regression	75.3	0.75
Linear discriminant analysis	76.0	0.76
K-neighbors	73.5	0.74
Decision Tree	80.5	0.80
Gaussian Naive Bayes	76.0	0.76
Support vector classification	71.5	0.71

The best result obtained in accuracy and ROC-AUC has been highlighted in orange. *ROC-AUC* Area under curve at receiver operating characteristics analysis

## Discussion

The main finding of our study is the presence of altered FC in AKP patients during rest, compared with matched controls, in brain areas associated with pain dimensions (prefrontal cortex, insula, thalamus, somatosensory cortex). Moreover, significant differences in FC were found in AKP patients showing catastrophizing compared with those without catastrophizing ideas. Therefore, etiopathogenesis of pain in AKP patients might not be limited exclusively to the knee, but also to underlying alterations in the central nervous system. In short, AKP should not be considered as a single musculoskeletal pathology. In this way, cortical changes might lead a perpetuation of pain.

Previous studies found nodes involved in the development of chronic pain in the DMN, SMN, CEN, and SN. In our study, the anterior insula, an important area of the SN, shows decreased correlation with the rostral prefrontal cortex, an area that is also part of the SN, and with the superior frontal gyrus, an area of the CEN. The insular cortex is often activated bilaterally during noxious somatosensory stimulation and has been suggested to play an important role in pain processing [27]. Afferent nociceptive information is transmitted rostrally from the second somatosensory cortex, SII, to the posterior insula and then to the anterior insula. Anterior insula activity may also modulate prefrontal cortex and anterior cingulate cortex activation in a task- or situation-dependent manner [28]. These findings suggest that the insula may be well positioned to use cognitive information to modulate connected brain areas involved in processing the sensory-discriminative, affective, and cognitive-evaluative components of pain. As part of the SN, we see that anterior insula acts on modulating the switch between the internally directed cognition and the externally directed cognition, activating de CEN and deactivating the DMN. The role of insula correlation with the prefrontal cortex in chronic pain has not yet been clarified, although most studies found that, as with other cortical areas, reduced correlation of the insula with the prefrontal cortex is associated with increased pain. Compared with controls, patients with painful knee osteoarthritis showed reduced FC between the right anterior insula and other areas of the pain network, related to the frontal pole [29]. However, some studies report that increased correlation of the insula with the prefrontal cortex is also associated with increased pain in other chronic pain conditions, such as rheumatoid arthritis [30]. In the study of brain connectivity in painful knee osteoarthritis [29], the authors observed reduced correlation between the right anterior insula and the superior frontal gyrus, which is consistent with our results.

The rostral prefrontal cortex is involved in a large number of cognitive processes and executive functions such as prospective and working memory. It has been related to the medial frontal gyrus in prospective memory tasks [31]. Thus, the increased activation seen in AKP patients may be due to their impaired prospective memory, as has been seen in other forms of chronic pain [32]. Moreover, we see that regional homogeneity, a parameter related with brain local activation, is significantly increased in the bilateral middle frontal gyrus and decreased in the left insula and superior frontal gyrus in patients with chronic neck and shoulder pain [33]. This supports the idea that there are differences in the behavior of the superior frontal gyrus and medial frontal gyrus regions in the condition of chronic pain despite the fact that both regions belong to the CEN.

On the other hand, previous studies [34] have shown that the role of the cerebellum seems to be very important in the development of chronic pain, since there are alterations in the correlation of the cerebellum with multiple regions. This region is considered important for sensorimotor and postural control as it controls the acquisition of sensory signals on which the motor system depends [35].

In this study, we found a decreased correlation of the anterior cerebellar network with the upper motor area, a crucial region for proper motor planning. Previous studies in chronic low back pain condition showed decreased resting-state brain activity in S1, supplemental motor area, M1 and cerebellar lobules IV-V for those subjects suffering from pain. This indicates that under the condition of chronic pain there is an impaired motor response to painful stimuli, which may indicate that chronic pain patients are overresponsive to sensory inputs that potentially signal danger to the body, thereby inducing maladaptive overgeneralized motor responses [36].

Connectome differences between different subsets of AKP patients (catastrophizing *versus* non-catastrophizing) has been demonstrated. This could explain the great variability in the pain experience among different subgroups of AKP patients. Pain catastrophizing is a psychological construct that includes cognitive, emotional and behavioral processes (fear-avoidance behaviors, altered mood and motivation) that amplify perceived painful sensations and predispose to the perpetuation of pain [37].When studying this effect in AKP patients, significant differences were found in the FC of AKP patients showing catastrophizing. Patients with catastrophizing showed, in general, an increase in correlation between different regions, especially in regions of the DMN, CEN, and cerebellum. It has previously been shown that catastrophizing scores are related to increased brain activity in regions such as the cerebellum, the medial frontal gyrus, regions of the descending pain modulation system such as dorsolateral prefrontal cortex or regions related to pain perception such as the thalamus or insula. Showing that, pain catastrophizing might be related to enhanced FC among areas playing a role in pain perception or between the DMN and descending pain modulatory system, also it could be related to sensorimotor perception [37]. It has been shown that thalamus has an important role in the modulation of nociception in neuropathic pain. It is involved in the descending inhibition to modulate nociceptive inputs. Moreover, it mediates different sensory discriminative and affective-motivational components of pain [38].

The potential of resting-state fMRI signatures to classify patients with chronic pain and healthy controls has been explored in this study. Significant differences were found in the analysis of fMRI images, enabling the creation of a classifier with an accuracy of 80.5%. While these results offer promise, it is essential to consider other confounding factors, such as medication usage, psychological changes, and plastic changes due to pain, to ensure their reliability in clinical settings. Given the importance of addressing confounders, external validation is necessary to assess the reproducibility of these findings. It is crucial to note that they cannot be solely relied upon as an accurate diagnosis for the presence of chronic AKP. However, they do provide valuable insights that can contribute to an enhanced understanding of the underlying mechanisms of pain. Physicians can benefit from these results as long as they are interpreted alongside other relevant factors to ensure a comprehensive evaluation of chronic pain conditions [39].

Despite the wide impact that AKP has in today’s society, there are very few studies that focus on this specific area to study the implications that chronic pain has on brain connectivity. This study is in line with a previous one [40], which also shows changes in FC in sensorimotor regions, insular cortex, and bilateral cerebellum, showing that the key to understand chronic AKP can be found in a hypoconnectivity between regions related to pain perception and sensorimotor control. This hypothesis is in line with a previous study [41] on brain activity during lower limb joint movement, in which it is determined that mainly the sensorimotor network and cerebellar-related areas are activated during this movement. To determine the specific differences between the various mechanisms of chronic pain, future studies comparing these conditions are needed. In order to determine the specific differences between the different mechanisms of chronic pain, future studies comparing these conditions are needed.

The findings of our research have important clinical implications that could have a significant impact on the diagnosis, treatment, and management of APK. By better understanding the FC patterns present in patients with anterior knee pain health care professionals could improve diagnostic accuracy. A deeper understanding of the brain networks and regions involved in APK could help identify specific subtypes of patients and allow for a more personalized approach to treatment.

Furthermore, by identifying the brain connections and areas that exhibit differences in APK, treatments targeting these specific regions could be more effective and lead to a significant reduction in pain and an improvement in patients’ quality of life. Also, knowledge of the brain networks associated with this disease could open new opportunities for the implementation of non-pharmacological therapies, such as non-invasive brain stimulation or biofeedback therapy, as possible complementary treatment options.

In the field of pain management, these findings could change the way APK is approached. By understanding that it is not just a phenomenon localized to the knee, but involves brain networks and neural connections, therapeutic approaches could be expanded to address both peripheral and central aspects of pain. This could result in more holistic and effective strategies for long-term management of APK.

A major limitation of this study is its cross-sectional methodological design, as it does not track individuals before the onset and through the development of pain. Consequently, the observed changes in FC correlations cannot be definitively attributed solely to the presence of chronic pain. Additionally, the relatively small number of patients and control subjects in our study raises concerns. However, based on a previous study on minimum sample size [42], and considering an effect size of 0.15 and a minimum acceptable ROC-AUC of around 0.70, our sample size of 45 subjects is reasonably close to the recommended range of 38 to 46 subjects.

While we believe that the observed effects are substantial enough to compensate for the small sample size, it is evident that larger samples are necessary due to the considerable variability in symptomatology among these patients. To strengthen the study validity, demographic variables showing significant differences were included as covariates to ensure that the observed changes were predominantly associated with AKP.

To maintain control over the error rate, a threshold of *p*-FDR ≤ 0.05 (uncorrected *p* values of ≤ 0.001) was applied, enhancing the consistency and robustness of the results. However, it is crucial to recognize that FC measures provide valuable insights into brain networks related to AKP but do not offer definitive causal explanations. A more comprehensive approach, considering additional relevant factors such as pain intensity or duration, and incorporating longitudinal studies, would be necessary to establish a clearer understanding of the role of FC in the etiopathogenesis of pain in AKP patients.

Lastly, it is essential to acknowledge that pain is a complex and multifaceted phenomenon influenced by various biological, psychological, and environmental factors. Relying solely on FC to explain the entire etiology of pain in these patients may oversimplify the underlying mechanisms. A comprehensive and integrative approach is needed to grasp the full complexity of AKP etiopathogenesis.

In conclusion, our data support the general idea that chronic pain has a disruptive effect on the functional brain network. Our study demonstrates that FC is altered in patients with AKP compared to matched control subjects, having a generalized impact on global brain function. Furthermore, it is shown that depending on the level of catastrophizing thoughts presented by the patients, new alterations in FC appear. It remains to be determined whether these extremely variable symptoms of AKP are related to fMRI imbalance, although the present study is a first step in that direction. The use of the results of brain alterations in a clinical decision support system could help the clinician to identify this disease, which is mostly diagnosed by discarding others. This could be a first step towards the objectification and identification of pain.

## Data Availability

Data used in this study are of a private/confidential nature and will not be available for public access due to privacy and confidentiality restrictions.

## Abbreviations

AKP: Anterior knee pain
BOLD: Blood oxygenation level-dependent
CEN: Central executive network
CN: Cerebellar network
DAN: Dorsal attention network
DMN: Default mode network
FC: Functional connectivity
FDR: False discovery rate
MNI: Montreal Neurological Institute
ROC-AUC: Area under curve at receiver operating characteristics analysis
ROI: Regions of interest
SMN: Sensorimotor network
SN: Salience network
VAS: Visual analog scale
VN: Visual network
